# Workplace bullying and sexual harassment among general surgery residents in Colombia

**DOI:** 10.7705/biomedica.6915

**Published:** 2023-06-30

**Authors:** Luis Carlos Domínguez, Lilian Torregrosa, Liliana Cuevas, Laura Peña, Sebastián Sánchez, Mauricio Pedraza, Álvaro Sanabria

**Affiliations:** 1 Departamento de Cirugía, Universidad de la Sabana, Chía, Colombia Universidad de la Sabana Universidad de la Sabana Chía Colombia; 2 Departamento de Cirugía, Pontificia Universidad Javeriana, Bogotá, D. C., Colombia Pontificia Universidad Javeriana Pontificia Universidad Javeriana Bogotá D. C Colombia; 3 Departamento de Cirugía, Universidad El Bosque, Bogotá, D. C., Colombia Universidad El Bosque Universidad El Bosque Bogotá D. C Colombia; 4 Departamento de Cirugía, Universidad de Antioquia, Medellín, Colombia Universidad de Antioquia Universidad de Antioquia Medellín Colombia

**Keywords:** Occupational stress, sexual harassment, medical staff, hospital, crosssectional studies, Acoso laboral, acoso sexual, discriminación, cirugía, personal médico hospitalario, estudios transversales

## Abstract

**Introduction.:**

Workplace bullying and sexual harassment are concerns among general surgery residents in Colombia.

**Objective.:**

To explore the prevalence and impact of workplace bullying and sexual harassment incidents among general surgery residents in Colombia.

**Materials and methods.:**

This nationwide study was conducted in 2020. Residents selfrated their exposure to workplace bullying and to sexual harassment in the forms of gender harassment, unwanted sexual attention, and sexual coercion. We analyzed demographic variables, perpetrator’s characteristics, and differences between victims and non-victims.

**Results.:**

The study included 302 residents. It found that 49% of general surgery residents in Colombia suffered from workplace bullying and 14.9% experienced sexual harassment. The main forms of sexual harassment were gender harassment (47%) and unwanted sexual attention (47%). Women reported significantly higher rates of being sexually harassed.

Surgeons were the main perpetrators of sexual harassment.

**Conclusions.:**

Workplace bullying and sexual harassment are frequent events in general surgery residency in Colombia. These findings suggest the need for interventions to improve the educational culture of surgical departments and decrease the prevalence of these behaviors.

## Resumen

**Introducción:**

El acoso laboral y el acoso sexual son preocupaciones en la formación quirúrgica.

**Objetivo:**

Exploramos la magnitud de estos problemas entre los residentes de cirugía general en Colombia.

**Materiales y métodos:**

se realizó un estudio nacional en junio de 2020. Los residentes autoevaluaron su exposición a la intimidación y el acoso sexual en forma de acoso de género, atención sexual no deseada y coerción sexual. Se analizaron variables demográficas y perpetradores entre víctimas y no víctimas.

**Resultados:**

Se incluyeron un total de 302 residentes. Las tasas de acoso laboral y sexual fueron del 49% y 14,9%, respectivamente. Las principales formas de acoso sexual correspondieron al acoso de género (47%) y la atención sexual no deseada (47%). El acoso sexual fue significativamente mayor entre las mujeres. Los cirujanos fueron los principales perpetradores.

**Conclusiones:**

El acoso laboral y el acoso sexual son frecuentes en la formación quirúrgica en Colombia. Estos hallazgos conducen a intervenciones para mejorar la cultura educativa de los departamentos quirúrgicos para disminuir la prevalencia de estos comportamientos.

Workplace bullying and sexual harassment are serious problems in general surgery residency. Incidents of workplace bullying and sexual harassment occur worldwide. A recent systematic review revealed a global pooled prevalence as high as 63% for workplace bullying and 27% for sexual harassment ^1^. The consequences of these negative behaviors have been extensively explored. In a residency learning environment, workplace bullying and sexual harassment are problematic due to physical and psychological implications for the resident, such as burnout, depression, and post-traumatic stress disorder. They also affect the quality of education and patient safety ^2^^-^^8^. Unfortunately, despite their negative consequences, workplace bullying and sexual harassment events are rarely reported by victims ^9^^-^^11^. Lately, workplace bullying and sexual harassment are enabled by a poor educational and ethical cultures in surgical departments, conditions closely related to the hierarchical and competitive structure of surgical training ^12^^,^^13^.

Surgical training takes place in a working and learning environment. In this workplace, workplace bullying occurs in “situations where an employee is persistently exposed to negative and aggressive behaviors at work, primarily of a psychological nature, with the effect of humiliating, intimidating, frightening, or punishing the target” as defined by Smith-Han *et al*. ^14^.

According to the United States National Academy of Sciences, Engineering, and Medicine, sexual harassment represents a type of discrimination in the form of ^1^*Gender harassment,* described as “verbal and non-verbal behaviors that convey hostility, objectification, exclusion, or second-class status about members of one gender”. ^2^*Unwanted sexual attention*, described as “verbal or physical unwelcome sexual advances, which can include assault”, and ^3^*Sexual coercion,* described as “favorable professional or educational treatment... conditioned on sexual activity” ^15^.

Assessment of workplace bullying and sexual harassment in Latin America requires attention. The region offers as many as 300 general surgery residency programs, in countries like Brazil, Argentina, Mexico, and Colombia, among others ^16^^-^^18^. According to the Lancet Commission on Global Surgery, Latin America also holds a representative proportion of the global surgical workforce. The ratio of surgeons in the region ranges from 40-59.9 per 100,000 population ^19^. In the region, information on workplace bullying and sexual harassment is limited and studies on occurrences are conducted from limited perspectives. Few studies provide information from the perspective of active surgeons, and even fewer report information from the viewpoint of residents ^20^.

By focusing on the perceptions of general surgery residents, the present study provides evidence on the prevalence of workplace bullying and sexual harassment in Colombia. This study also responds to increasing societal calls for periodic screening ^22^ in an era of zero tolerance for workplace bullying and sexual harassment ^21^. This study may also potentially lead to customized interventions ^23^^-^^25^.

## Material and methods

### Research design, context, and participants

This cross-sectional, multi-institutional study took place in June, 2020. Colombia has 20 university-based general surgery residency programs distributed in four regions: Northern, Central, Western, and Southern. Programs in the Central Region have a surgical tradition based on the Halstedian model of training. Surgical training spans four years. The estimated number of categorical residents is 380. We invited all residents, regardless of age, sex, year of training, or type of program, to participate in this research.

### Measures and instruments

Residents self-rated their exposure to workplace bullying on a shortened version of the Negative Acts Questionnaire (NAQ) published in Spanish ^26^.

Using 14 questions, the NAQ assessed the frequency of workplace bullying in the prior six months, ranging from one (never) to five (daily). Additionally, residents rated the frequency for each negative behavior by different perpetrators (i.e., surgeons in charge of training, peers, patients) on a Likert scale. The scale ranged from one (extremely infrequent) to ten (extremely frequent). The coefficient of reliability of the questionnaire is 0.85.

To assess exposure to sexual harassment, researchers presented to participants the definition of the three forms of sexual harassment according to the United States National Academy of Sciences, Engineering, and Medicine ^15^. The authors asked if the residents were victims of any of those behaviors during the prior six months. For positive responses, participants rated the frequency of exposure on the Likert scale from one (never) to five (daily).

### Data collection

The Division of Education of the Colombian Association of Surgery, in cooperation with program directors, coordinated data collection. Participants received an online survey containing the questionnaire and asking for demographic information (program, age, sex, and year of residency training). Once the anonymous questionnaire was completed and returned, researchers coordinated data transcription and organization to assure confidentiality and integrity of the information.

### Statistical analysis

The authors analyzed the information when at least 60% of the available programs and 60% of the total number of active residents per program completed the questionnaires. Measurement of frequencies and descriptive statistics were used to gain insight into the relevant characteristics of the population.

For NAQ, a total score resulted from adding the scores given to each behavior by residents. Interpretation of the total score used percentiles. A NAQ score below the 25^th^ percentile was interpreted as “not bullied”, a score from the 26^th^-75^th^ percentile was considered as “occasionally bullied”, and participants who scored above the 75^th^ percentile were considered “victims of continuous workplace bullying”. The results present workplace bullying as percentages and ranges. Similarly, the mean scores, standard deviations, and 95% confidence intervals (CI) were computed for the frequency of workplace bullying per perpetrator based on the 1-to-10-point Likert scale.

For residents who rated any form of sexual harassment during the prior six months, the percentages of sexual harassment were computed, irrespective of its frequency (i.e., daily, monthly). Ultimately, univariate comparisons of the demographic variables between victims and non-victims of workplace bullying and sexual harassment were determined with the chi square test (p<0.05). The Stata 15™ software used for the statistical analyses.

### Ethical considerations

The study obtained individual informed consent from all participants. The Institutional Review Board of the Faculty of Medicine of Pontificia Universidad Javeriana gave ethics approval for the study (reference number: FM- CIE 084220).

## Results

A total of 302 residents from 20 general surgery residency programs in Colombia participated in the study, a 79.4% response rate. Table 1 shows the characteristics of the participants. Female residents made up 42. % of respondents. The mean age of participants was 28.9 ± 3.33 (23-42) years.

**Table 1:** Population characteristics associated with workplace bullying according to the shortened version of the Negative Acts Questionnaire

Characteristic		Total n (%)	Non-victim n (%)	Occasional WPB n (%)	Continuous WPB n (%)	p value
Residents		302	154 (51)	83 (274)	65 (21.5)	
Sex						NS
	Male	175 (579)	88 (50.2)	50 (28.5)	37 (21.1)	
	Female	127 (42.0)	66 (51.9)	33 (25.9)	28 (22.0)	
University						NS
	Public	96 (31.7)	51 (53.3)	30 (31.2)	15 (15.6)	
	Private	206 (68.2)	103 (50)	53 (25.7)	50 (24.2)	
Year of residency						NS
	1	81 (26.8)	45 (55.53)	20 (24.6)	16 (19.7)	
	2	91 (30.1)	43 (472)	29 (31.8)	19 (20.8)	
	3	73 (24.1)	32 (43.8)	23 (31.5)	18 (24.6)	
	4	57 (18.8)	34 (59.6)	11 (19.3)	12 (21.0)	
Region						0.001
	Central	164 (54.3)	66 (40.2)	56 (34.1)	42 (25.6)	
	Northern	52 (172)	39 (75)	9 (173)	4 (7.6)	
	Western	59 (19.5)	33 (55.9)	13 (22)	13 (22.0)	
	Southern	27 (8.9)	16 (59.2)	5 (18.5)	6 (22.2)	

WPB: workplace bullying; NS: non-significant

**Figure 1:**
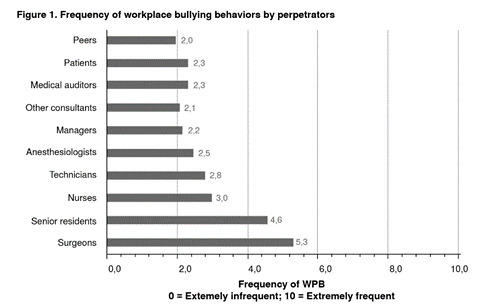
Perpetrators and frequency of workplace bullying

The global rate of workplace bullying was 49% (148/302). Occasional workplace bullying occurred in 27.4% (83/302) and continuous workplace bullying in 21% (65/302) of the cases. There were no differences between victims and non-victims of workplace bullying based on sex, type of program (private vs. public), or year of residency training. However, we found significant differences by geographical distribution of programs (Table 1). Of the 164 residents in programs of the Central Region, 80 (59.7%) experienced workplace bullying, a much higher percentage than those from other regions (Northern: 25%; Western: 44%, and Southern: 40.7%). The most frequent negative behavior was “being shouted at or being a target of spontaneous rage” (67.5%). Table 2 presents the absolute frequencies of negative behaviors. The main perpetrators of workplace bullying in Colombia were surgeons in charge of training, 5.31 ± 2.97 (Likert scale: 1-10). Figure 1 presents negative behaviors by perpetrators.

**Table 2:** Frequency of negative behaviors according to the shortened version of the Negative Acts Questionnaire

Negative behaviors		Total n (%)	Never n (%)	Sometimes n (%)	Monthly n (%)	Weekly (%) n	Daily n (%)
1	Being shouted at or being the target of spontaneous anger	204 (675)	98 (32.4)	141 (46.4)	35 (11.5)	25 (8.2)	3(0.9)
2	Being ordered to do work below your level of competence	202 (66.8)	100 (33.1)	129 (42.7)	28 (9.2)	32 (10.6)	13 (4.0)
3	Having your opinions and views ignored	202 (66.8)	100 (33.1)	150 (49.6)	27 (8.9)	19 (6.2)	6 (1.9)
4	Persistent criticism of your errors and mistakes	201 (66.5)	101 (33.4)	141 (46.4)	21 (6.9)	29 (9.6)	10 (3.3)
5	Being humiliated or ridiculed in connection with your work	200 (66.2)	102 (33.7)	141 (46.6)	26 (8.6)	26 (8.6)	7(2.3)
6	Spreading of gossip and rumors about you	184 (60.9)	118 (39.0)	120 (39.7)	32 (10.6)	15 (4.9)	17 (5.6)
7	Being exposed to an unmanageable workload	171 (56.6)	131 (43.3)	132 (43.7)	16 (5.3)	17 (5.6)	6 (1.9)
8	Having insulting or offensive remarks made about your person (i.e., habits and background), your attitudes, or your private life	158 (52.3)	144 (47.6)	113 (374)	22 (7.2)	20 (6.6)	3(0.9)
9	Someone withholding information that affects your performance	157 (51.9)	145 (48.0)	103 (34.1)	25 (8.2)	24 (7.9)	5 (1.6)
10	Excessive monitoring of your work	141 (46.6)	161 (53.3)	99 (32.7)	18 (5.0)	18 (5.9)	6 (1.9)
11	Being ignored or excluded	125 (41.3)	177 (58.6)	94 (31.1)	16 (5.3)	13 (4.3)	2 (0.6)
12	Pressure not to claim something you are entitled to by right	115 (38.08)	187 (61.9)	77 (25.5)	19 (6.2)	13 (4.3)	6 (1.9)
13	Intimidating behavior such as finger-pointing, invasion of personal space, shoving, blocking/barring the way	46 (15.2)	256 (84.7)	30 (9.9)	12 (3.9)	2 (0.6)	2 (0.6)
14	Threats of violence or physical abuse or actual abuse	20 (6.6)	282 (93.3)	14 (4.6)	2 (0.6)	3 (0.9)	1 (0.3)

WPB: workplace bullying

The global rate of sexual harassment was 14.9 (45/302 cases). A total of 21 cases (47%) corresponded to gender harassment, 21 cases (47%) to unwanted sexual attention, and 3 cases (7%) to sexual coercion. The study found no differences between victims of and non-victims of sexual harassment according to the type of program (private vs. public) or year of residency training. There were significant differences between males and females (4.5% vs. 29.1%, p<0.05, respectively), and among geographical regions. Of the 45 cases in the country, 27 (60%) occurred in the Central region (p<0.05) (table 3).

**Table 3:** Characteristics of the population associated with sexual harassment

Characteristic		Total n (%)	Non-victim n (%)	Victim n (%)	p value
Residents		302	257 (85.1)	45 (14.9)	
	Sex				0.001
	Male	175 (579)	167 (95.4)	8 (4.57)	
	Female	127 (42.0)	90 (70.8)	37 (29.1)	
University					
	Public	96 (31.7)	82 (85.4)	14 (14.5)	NS
	Private	206 (68.2)	175 (84.9)	31 (15.0)	
Year of residency					NS
	1	81 (26.8)	70 (86.4)	11 (13.5)	
	2	91 (30.1)	71 (78.0)	20 (21.9)	
	3	73 (24.1)	64 (876)	9 (12.3)	
	4	57 (18.8)	52 (91.2)	5 (8.7)	
Region					0.04
	Central	164 (54.3)	137 (83.5)	27 (16.4)	
	Northern	52 (172)	50 (96.1)	2 (3.8)	
	Western	59 (19.5)	49 (83.0)	10 (16.9)	
	Southern	27 (8.9)	21 (777)	6 (22.2)	

NS: non-significant

## Discussion

This study found that 49% of residents in Colombia experienced workplace bullying, and 14.9 % were victims of sexual harassment in 2020. Females were the most frequently victimized, with surgeons in charge of training as the main perpetrators. There were no differences in the negative behaviors related to the year of residency training. The predominant behavior related to workplace bullying was “being shouted at or being a target of spontaneous rage.” Unwanted sexual attention and gender harassment were the most frequent forms of sexual harassment.

For general surgery residents, Gianakos *et al.* reported global pooled rates of workplace bullying at 63% and sexual harassment at 27% ^1^. The present study reports a lower rate of occurrence of workplace bullying and sexual harassment in Colombia. However, the prevalence of the problem is higher in Colombia than in Spain. A NAQ-based study in Spain reported a 37.7% incidence of workplace bullying ^27^. Despite data discrepancy, some patterns of the problem in Colombia are like patterns in other countries. For example, as in other studies, workplace bullying and sexual harassment were significantly higher among females. Also, surgeons in charge of training were the most likely perpetrators ^21^^,^^28^^-^^33^.

The rates of workplace bullying and sexual harassment were higher among programs located in the Central region of Colombia. The training there is characterized by a long-standing tradition of hierarchies and competitiveness. These findings are consistent with previous studies reporting that the educational culture influences workplace bullying and sexual harassment in surgical training ^12^^,^^13^.

Results in the present study point to unwanted sexual attention and gender harassment as the most common forms of sexual harassment. Some studies describe these as crude, demeaning, explicit comments and/or racist or homophobic attitudes ^33^^,^^34^. In some reports, the problem seems more a concern for junior residents ^2^, but findings of this study do not support this observation. The results also differ in the type of workplace bullying attitudes. In other studies, the most frequent forms of workplace bullying, perceived by residents, were “ignoring the opinions”, “exposing to unmanageable workload”, and “reminding mistakes” ^11^^,^^35^.

This is the first study reporting workplace bullying and sexual harassment among surgical trainees. Some other studies available in Latin America described workplace bullying and sexual harassment prevalence only among practicing surgeons. A study in Ecuador reported a rate of 55.2% sexual harassment among practicing women surgeons ^20^. Similarly, a study in Mexico reported rates of 84% of workplace bullying and 24 % of sexual harassment in neurosurgery residents ^36^. This study included more than 90% of the general surgery residents in Colombia. This percentage ensured a thorough exploration of workplace bullying and sexual harassment in the country.

Even so, this study has a few limitations. The study asked for residents’ perceptions six months prior to filling out questionnaires; as the study was carried out four months after the onset of COVID-19 pandemic, the authors acknowledge the possible influence of the pandemic on the study results. Also, the study lacks qualitative perspective, such as voices of residents and surgeons in charge of training, to explore the main findings in greater depth. Ultimately, the study does not address the consequences of workplace bullying and sexual harassment for residents, institutions, and patients. These limitations call for future research. Longitudinal studies are also needed to compare the prevalence of workplace bullying and sexual harassment to the baseline measures of this study. Additionally, mixed methods research can investigate the dynamics of this complex problem. New studies on the consequences of workplace bullying and sexual harassment for different stakeholders and the educational culture would be welcome.

In response to growing concerns about workplace bullying and sexual harassment, this study also supports practices that strive for a healthy educational culture in surgery. The participant institutions received a report of baseline measures describing the magnitude of workplace bullying and sexual harassment in each program.

Similarly, in meetings of the CAS Division of Education, the authors discussed the study’s key findings with the directors of the residency programs. The authors anticipate that feedback and regular measures will help programs reflect on and appropriately react to this problem.

Finally, the findings of this study can create awareness about workplace bullying and sexual harassment among residents. Considering that residents are often victims, but also sometimes perpetrators of these behaviors, we call for their heightened awareness as a potential solution to the problem. Residents must speak up and report these unethical behaviors and promote a candid social discussion about them ^1^^,^^23^^,^^24^. We fervently hope this study contributes to the design of strategies to diminish workplace bullying and sexual harassment. These strategies should focus on transforming the educational culture of residency and medical practice and promoting healthy climates for learning.

In conclusion, workplace bullying and sexual harassment continue to be a serious, persistent, and widespread problem in general surgery training. This study describes the prevalence of workplace bullying and sexual harassment and its associated characteristics in Colombia. This is a Latin American country with a long tradition of surgical training at high standards, though not without its problems. The authors provide these findings to the existing body of evidence on workplace bullying and sexual harassment around the world. This study has implications for current practice and other future research.
